# 

**Published:** 1819-10-01

**Authors:** 


### CORRESPONDENCE.

The Review of a recent medical work, enclosed in a blank sheet of paper, has been received by the twopenny post. This being rather an unusual and suspicious phenomenon, we took pains to carefully compare the critique with the work criticised, and we acknowledge that it is an able and impartial Review. Yet we cannot insert it, from the circumstance of its being *anonymous*; but if the writer will confide his name to us, it shall appear *verbatim.* If nothing further is heard before the 15th of November, we shall consider ourselves at liberty to appropriate such parts as suit us, in our analysis of the work alluded to. In *this* way, viz. with the writer's name confidentially communicated, we shall be thankful for *critical remarks* on works, or on parts of works, the Editor pledging himself to examine all such contributions with the most scrupulous attention, lest any *improper or unfair criticism* should tarnish the pages of the Medico-Chirurgical Journal. Indeed, the Editor flatters himself that his character and responsibility will be a sufficient guarantee to the Public, that no improper transactions shall take place behind the scenes. He has too much at stake to risk such things, even had he no sense of honour or conscience. But, at the same time, he will give every scope and facility to *talent,* provided it be guided by candour, impartiality, and strict justice.

*** The back numbers of the Journal having been reprinted, at a very great expence, it is requested that gentlemen will complete their sets as soon as possible. Some of the reprinted numbers are already getting low; and when out of print again, no new edition will ever be published, as a *second* Quarterly Series of the Journal will then commence.

The following works have been received for Review, and shall have precedence in publication: — Dr. Scudamore's Third Edition on Gout; Dr. Adam Hunter's Essay on the Harrowgate Waters; Dr. Black's Pathological Essays; Dr. Armstrong's Third Edition on Typhus; Mr. Wilson's Lectures delivered at the College of Surgeons; Dr. Hall on the Mimoses; Mr. Curtis on the Physiology and Diseases of the Ear; together with some others, which will appear in the next number.

The rule will be as nearly general as possible, that works shall be reviewed in the order of their transmission to the Managing Editor.

### COMMUNICATIONS

Have been received from Mr. Webster, 51st Regiment; Dr. Dickson, of Clifton; Dr. Lucas, of Hatfield; Mr. Dungleson, of Prescott-street; Dr. Foster, of Oakes; Mr. George Williams, of Portsea; Dr. James Kennedy, of Dunning; Dr. Felix, of Bristol; Mr. Shoveller, of West Chapel; Dr. James Clark, Resident English Physician at Rome; Dr. Fisher, of Bath; Mr. Gideon Mantell, Surgeon to the Royal Ordinance Hospital, Ringmer; Dr. J. B. Davis, Physician to the Universal Dispensary for Children; Mr. Walter C. Dendy, Surgeon to the Universal Dispensary for ditto.

From such Communications as are *voluminous,* (and many are such) short extracts will be made, that may not, in the least, interfere with their publication entire, through some other medium; while the shorter Communications will have place in the Review Department, as soon as convenient, with little or no curtailment. It is not possible for the Editor, in any other way, to accommodate his numerous Correspondents, and do justice to the great interests of medical science, in the Review Department. As all must sacrifice something for the general good, it is to be hoped that the Editor's endeavours will be judged of with indulgence in cases like this, where it is so extremely difficult to meet the views and wishes of every individual.

"Time (says Lord Bacon) moveth so round, that a froward retention of custom is as turbulent a thing as an innovation. Surely every medicine is an innovation; and he that will not apply new remedies, must expect new evils—for TIME is the greatest innovator; and if Time, of course, alter things to the worse, and wisdom and counsel shall not alter them to the better, what shall be the end?"

*Essay on Innovations.*

### *Additional Patronage since last Quarter.*

Baron, Dr. John, Gloucester
Bartley, Dr. Assistant Surgeon to the Forces
Bath, Dr. Sen, King street, Portman-square
Beatty, William, M. D F. R. S. &c. Physician of the Fleet, &c, London
Belcombe, Dr. H. S. Newcastle-under-Line, Staffordshire
Blackett, Charles Powel, Park Street, Grosvenor square
Blake, Mr. Chemist, Piccadilly
Broadfoot, Dr. Inspector General of Health, Corfu
Burnidge, Mr. T. D, Worcester
Buxton, J. Esq. Gatton Park, Ryegate
Cary, Mr. J. W. Surgeon, Trowbridge
Chatham Medical Society
Clark, Dr. James, Resident Physician, Rome
Copland, Dr. 2, Walworth Terrace
Curry, Dr.
Davidson, Dr. Nottinghamshire
Davis, Mr. William, 33, Orchard-street, Portman square
Denecke, Dr. Isle of Wight
Downe, Dr. J. S. Resident Physician, Florence
Ducamp, M. Docteur en Medecine, Rue St, Martin, à Paris
Dugdale, Mr. Blackburn, Lancashire
Elliot, James, Esq. Surgeon to the Forces
Farnden, Joseph, Esq. Surgeon, 70th Regiment, Canada
Foster, Dr. William, Member of the Royal Medical Society, Edinburgh, Oakes, near Sheffield
Gronous, Mr. West Indies
Hands, Mr. B. Turnham Green
Harshaw, Mr. Surgeon, R. N.
Hillier, Mr. H. Dean street, Borough
Hora, Mr. James, Surgeon, Harwich
Hull, Dr. Manchester
Jenkins, Mr. Fossey, Surgeon, Sutton Valence, near Maidstone, Kent
Johnston, Dr. Ireland
Jones, Dr. George Haines, Portsmouth
Jouenne, Dr. Rue de L'Orangerie, Brussels
Kerr, Robert, Esq. Porto Bello, South America
Lucas Dr. C. E. Hatfield
M'Whirter, Dr. F. Newcastle-upon-Tyne
M'Dougle, Dr. Paris
Magrath, George, M. D. F. R. S. and L. S. Physician to the Dock and Stonehouse Dispensary, Plymouth
Mantell, Gideon, Esq. F. L. S. Surgeon to the Royal Ordinance Hospital at Ringmer, Castle Place, Lewes
Marshall, Mr. Surgeon, Belfast
Parmly, Mr. Surgeon Dentist, Pall-Mall
Phillips, Mr. Samuel, H. M. S. Nimrod, Leith
Pierce, Dr. Edward, Wexford
Reilly, J. Esq. Plymstock, near Plymouth
Ring, John, Esq. New-street, Hanover-square
Robinson, Mr. Holles-street, Cavendish square
Robinson, Mr. Mark, Beverley, Yorkshire
Roberton, James, Esq. Hospital Assistant to the Forces
Russell, Mr. R.
Scalt, Mr. Surgeon, Easinwold, Yorkshire
Scott, Dr. Russell-square
Shearman, Dr. William, Northampton-square
Slow, D. Esq. Royal Horse Guards
Smart, Mr. Frith-street, Soho
Stenson, Dr. Burton on Water, 2 Copies
Stollerforth, —— Esq. Boulton-street, Piccadilly
Thomson, Dr. Paris
Walton, Mr. J. E. Surgeon, Guilford-street
Wilson, James, Esq. F. R. S. George Street, Hanover-square
Wilson, Mr. Surgeon, Belfast
Wilmore, Edmund William, Member of the Royal College of Surgeons in London, Paris
Williamson, Dr. Whitehaven
York Hospital Library

☞ What is meant by a *Subscriber* to this Journal is merely he who takes the Journal in, through his own Bookseller, the same as any other periodical work. A complete alphabetical List will accompany the next Index, and be regularly republished with each Volume, with such Additions or Alterations as each Year may make. Not more than a third of the actual Purchasers have yet transmitted their Names. This List and Work will form a curious record, on some future occasion, of the contemporary Authors and Practitioners of the present enlightened Æra.

